# Editorial Note

**Published:** 1934

**Authors:** 


					Editorial Note
Liverpool
Medical School
Centenary.
The Centenary of the Foundation
of the Liverpool Medical School
was celebrated on Friday, 11th May,
by a large concourse of former
students and graduates of the
University and delegates from other Universities
and the Medical Corporations. The sisterly greetings
and congratulations of Bristol University were con-
veyed by Professor E. Fawcett. The story of the
foundation and progress of the School has been told
for us by Dr. Gemmell,* who well describes the modest
beginnings and early struggles, not unlike those which
our own School encountered. From these has emerged
a School of Medicine known all the world over for its
thoroughness and its enterprise, particularly, of course,
for the pioneer work of the School of Tropical Medicine.
On behalf of the Bristol Medico-Chirurgical Society
we tender our sincere good wishes and heart-felt
congratulations on this auspicious occasion.
* The Liverpool Medical School, 1834-1934. Dr. A. A. Gemmell.
London : Hodder and Stoughton Ltd. Price Is.

				

